# Prediction of Recurrent Mutations in SARS-CoV-2 Using Artificial Neural Networks

**DOI:** 10.3390/ijms232314683

**Published:** 2022-11-24

**Authors:** Bryan Saldivar-Espinoza, Guillem Macip, Pol Garcia-Segura, Júlia Mestres-Truyol, Pere Puigbò, Adrià Cereto-Massagué, Gerard Pujadas, Santiago Garcia-Vallve

**Affiliations:** 1 Research Group in Cheminformatics & Nutrition, Departament de Bioquímica i Biotecnologia, Campus de Sescelades, Universitat Rovira i Virgili, 43007 Tarragona, Spain; 2 Department of Biology, University of Turku, 20500 Turku, Finland; 3 Department of Biochemistry and Biotechnology, Rovira i Virgili University, 43007 Tarragona, Spain; 4 Nutrition and Health Unit, Eurecat Technology Centre of Catalonia, 43204 Reus, Spain; 5 EURECAT Centre Tecnològic de Catalunya, Centre for Omic Sciences (COS), Joint Unit Universitat Rovira i Virgili-EURECAT, Unique Scientific and Technical Infrastructures (ICTS), 43204 Reus, Spain

**Keywords:** SARS-CoV-2, COVID-19, machine learning, mutations

## Abstract

Predicting SARS-CoV-2 mutations is difficult, but predicting recurrent mutations driven by the host, such as those caused by host deaminases, is feasible. We used machine learning to predict which positions from the SARS-CoV-2 genome will hold a recurrent mutation and which mutations will be the most recurrent. We used data from April 2021 that we separated into three sets: a training set, a validation set, and an independent test set. For the test set, we obtained a specificity value of 0.69, a sensitivity value of 0.79, and an Area Under the Curve (AUC) of 0.8, showing that the prediction of recurrent SARS-CoV-2 mutations is feasible. Subsequently, we compared our predictions with updated data from January 2022, showing that some of the false positives in our prediction model become true positives later on. The most important variables detected by the model’s Shapley Additive exPlanation (SHAP) are the nucleotide that mutates and RNA reactivity. This is consistent with the SARS-CoV-2 mutational bias pattern and the preference of some host deaminases for specific sequences and RNA secondary structures. We extend our investigation by analyzing the mutations from the variants of concern Alpha, Beta, Delta, Gamma, and Omicron. Finally, we analyzed amino acid changes by looking at the predicted recurrent mutations in the M-pro and spike proteins.

## 1. Introduction

SARS-CoV-2 is the coronavirus that causes COVID-19. It has a positive sense single-stranded RNA (ssRNA) genome of around 29,900 nucleotides that codifies 11 genes: ORF1ab, spike (S), ORF3a, envelope (E), membrane (M), ORF6, ORF7a, ORF7b, ORF8, nucleocapsid (N), and ORF10 [1,2]. The ORF1ab gene encodes the polyproteins pp1a and pp1ab, which are further cleaved by the main protease (M-pro) and papain-like protease (PLpro) [3]. Pp1ab includes pp1a, and its synthesis requires a ribosomal frameshift [3]. When pp1ab cleaves, it gives rise to 15 proteins: a lead protein, nsp2, nsp3 (PLpro), nsp4, nsp5 (M-pro), nsp6, nsp7, nsp8, nsp9, nsp10, nsp12 (an RNA-dependent RNA polymerase, RdRp), nsp13 (a helicase), nsp14 (a 3′-5′ exonuclease), nsp15 (an endoRNAse) and nsp16 (a 2’-O-ribose methyltransferase) [3].

Like other viruses, the SARS-CoV-2 genome mutates. Mutations can lead to enhanced viral fitness and the emergence of virus variants [4]. However, recombination and reassortment are also important mechanisms to generate genomic variability [5]. Virus mutation rates vary widely [6], but coronaviruses have a proofreading activity (due to the nsp14 gene) [7] that may explain their abnormally large genome compared to other ssRNA viruses [5]. Mutations can be caused by RNA polymerase errors during virus replication or by the deamination of unpaired nitrogenous bases caused by host deaminases [6,8,9,10]. In mammalian species, apolipoprotein B mRNA editing catalytic polypeptide-like (APOBEC) enzymes deaminate cytosines into uracils (C > U) in single-stranded DNA (ssDNA) and ssRNA [11]. Recent experiments have demonstrated that APOBEC3A, APOBEC1, and APOBEC3G can effectively cause C > U mutations in the SARS-CoV-2 genome at specific sites [12]. Cytosines in UC and AC motifs showed the highest mutation rate, modulated by features of the RNA structure around these motifs [12]. This is consistent with previous results [13,14]. For example, the 5′-[U|A]C>U mutation occurs more frequently than 5′-[C|G]C > U (*p* = 0.0501) in the SARS-CoV-2 genome [14]. If APOBEC enzymes were to act on the negative strand of the SARS-CoV-2 genome, it would be reflected on the positive strand as G>A mutations [15]. Adenosine deaminases acting on RNA (ADAR) deaminate adenines into inosines (A > I) in double-stranded RNA (dsRNA) [16]. As inosine preferentially base pairs with cytidine, A > I mutations cause A > G and U > C transitions on the positive strand of the SARS-CoV-2 genome [15,17]. Most SARS-CoV-2 mutations are expected to be neutral, but some may be advantageous or deleterious to the virus [18]. Viruses experience selection pressure from their host’s immune system, defense mechanisms, antiviral drugs, and vaccines [5]. Highly deleterious mutations, such as those that prevent the virus from invading the host, are unlikely to be observed [18]. The high frequency of some mutations is not always due to an advantageous mutation. It can also be caused by a founder effect, which is when a mutation emerges early in the evolution of a pandemic and is transmitted to all of its descendants [19] or when they are found in a variant that carries an additional advantageous mutation.

During the COVID-19 pandemic, the number of new SARS-CoV-2 variants, including the variants of concern (VoCs), has steadily increased [20,21]. VoCs are variants that exhibit increased transmissibility; more severe disease; significantly decreased neutralization by antibodies developed from previous infection or immunization; reduced efficacy of therapies or vaccines, or failures of detection at diagnosis [22]. Therefore, it is very important to understand the mutational patterns in the evolution of SARS-CoV-2 and to predict its mutations in order to devise better antiviral treatments [23]. Due to the random nature of these mutations, predicting SARS-CoV-2 mutations caused by replication errors can be difficult. However, it is feasible to predict mutations driven by the host, such as those caused by host deaminases [12]. These mutations are expected to be recurrent, i.e., to appear multiple times independently and be present in several SARS-CoV-2 lineages. In this paper, we use machine learning (ML) to predict recurrent mutations that will emerge repeatedly and independently as the virus adapts to humans [18,24]. Before the pandemic, ML was used extensively in biology [25,26,27,28], for example, to predict mutations of influenza A viruses by predicting which AA position will mutate [29] and to predict recurrent mutations in cancer [30]. ML has been used throughout the SARS-CoV-2 pandemic as a tool to assist vaccine development and predict epitope hotspots [31]; the binding affinity of antibodies to mutations in the spike RBD [32]; the binding affinity of chemical compounds as inhibitors against the M-pro protein [33,34]; the clinical disease severity based on the virus genome mutations [35]; the mutation rate of nucleotide substitution (e.g., A > T) [36]; the subsequent nucleotide given a sequence of the SARS-CoV-2 genome, and also given a pair of sequences to indicate the location of the changes [37]; the antibody escape mutations of the spike protein [38]; the spread of spike protein mutation, based on fold-change per country [39]; future domain-specific spike mutations [40]; anti-SARS-CoV-2 activities from molecular structure [41]; and many more [42,43]. In this article, we start by showing some descriptive statistics of SARS-CoV-2 mutations. We continue by defining recurrent mutations. We then use ML models to predict which positions of the genome will have a recurrent mutation, showing the performance metrics of the models and variables that are more important for the ML models. Subsequently, we extend our investigation to predict which mutations will become recurrent and how our work can be used with the variants of concern Alpha, Beta, Delta, Gamma, and Omicron. Finally, we analyze amino acid changes by looking at the predicted recurrent mutations in the M-pro and spike proteins, evaluated with recent data from 2022.

## 2. Results and Discussion

### 2.1. SARS-CoV-2 Mutation Description

**Figure 1:**
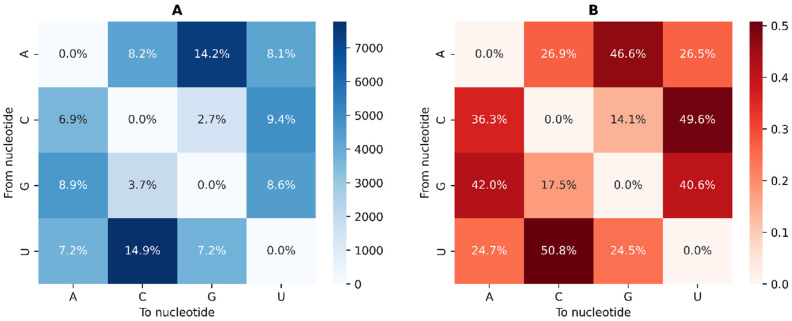
Nucleotide change count of unique single nucleotide mutations. (**A**) Total count of changes among unique mutations. (**B**) Nucleotide change normalized through the horizontal axis. Each row adds up to 1.

The GISAID database [44] had 877,086 SARS-CoV-2 genomes as of 19 April 2021. From these genomes, we found 25,353,899 mutations (including insertions and deletions), of which 52,160 were unique single nucleotide variants (SNVs) found in one or more genomes. Among the unique SNVs, adenine and uracil were the nucleotides with the most SNVs, 15,898 and 15,313, respectively (Figure 1A). Because the SARS-CoV-2 genome is richer in adenines and uracils (its G + C content is 37.97%), in SNVs, it is expected to find more adenines and uracils than guanines and cytosines. Transitions, i.e., U > C, A > G, C > U, and G > A, are more frequent than transversions (Figure 1B). When uracil mutates, 51% of the time, it mutates into a cytosine, with similar percentages found regarding other transitions (Figure 1B), with the exception of G > A, which has a slightly lower frequency (Figure 1B). C > G and G > C transversions are observed with lower frequencies (Figure 1B). Only 14% and 17% of cytosine and guanine SNVs are, respectively, C > G and G > C transversions. This may reflect the CpG avoidance that has been described for SARS-CoV-2 and other coronaviruses [45,46]. This CpG dinucleotide suppression is thought to be due to the fact that it is evading the zinc-finger antiviral protein (ZAP) that specifically binds CpG dinucleotides in ssRNA and causes its degradation [47,48].

### 2.2. Recurrent Mutations in SARS-CoV-2

Recurrent mutations (RM) are mutations that occur independently and many times throughout a virus’ evolution. They could be the result of host RNA-editing mechanisms or ongoing selection [18,24]. After analyzing 46,723 and 7710 SARS-CoV-2 genomes from July and April 2020, van Dorp et al. [18,24] identified 5710 and 198 RM, respectively. Among the RM, they found no evidence for increased transmissibility, suggesting that RMs were caused by RNA editing [18]. To identify RMs, van Drop et al. [18,24] used a multiple alignment and a maximum likelihood tree. However, due to the large number of analyzed sequences, we used another strategy. We used Pango nomenclature that classifies SARS-CoV-2 genomes into lineages [49,50]. The Pango nomenclature is a hierarchical and dynamic classification system based on phylogenetic evidence that uses ML to assign each SARS-CoV-2 genome to a lineage [49,50]. Although this system is not intended to represent every evolutionary change in SARS-CoV-2 [49], we have used it to count the number of different lineages in which each mutation is found. Taking advantage of the hierarchical nature of the Pango system, we then reduced this number by grouping related lineages and counting the number of distantly-related lineages (NDRL) (see Materials and Methods). This provided us with an estimate of the number of times a mutation emerged independently. Then, we defined RM for a set of NDRL thresholds of 5, 10, and 15. We used different NDRL thresholds to overcome potential sequencing errors, artefactual biases, and other causes, such as recombination, which may lead to homoplasies [14,24]. We found 22,738, 11,275, and 6767 RM for the 5, 10, and 15 NDRL thresholds. Dataset S1 contains all the mutations found and the number of NDRLs for each mutation. Mutations present in almost all Pango lineages that appeared early in the pandemic, such as the A23403G mutation that results in the D614G substitution of the spike protein, are not considered to be RM because they have an NDRL value of 1. As expected and based on previous work [14,18,46,51,52], RMs are rich in C > U mutations (Figure S1). For instance, for the NDRL threshold of 15, 47% of the 6767 mutations are C > U, while 19% are G > U. U > C, G > A, and A > G each constitute 10% (Figure S1).

### 2.3. Prediction of Whether a Given Position in the SARS-CoV-2 Genome Will Be Affected by a Recurrent Mutation

To predict whether a given position in the SARS-CoV-2 genome holds an RM, as defined by the NDRL thresholds of 1, 5, 10, and 15, a deep learning/machine learning model was trained using the artificial neural network/multi-layer perceptron architecture. The variables used to train the models were the SARS-CoV-2 genome sequence, the prediction of the secondary structure of the SARS-CoV-2 genome, the RNA normalized 2′-hydroxyl acylation analyzed by primer extension (SHAPE) reactivity [53], and the translated AA sequences of the coding parts of the genome. The genome variables were split into 13 position windows, with the central window position indicating the location of the possible mutation. The data split for the machine learning setup included a group of 16 genes for training and four different genes for validation (Figure S2). To evaluate the model predictions, a separate test set was used. The test set was not used at any moment during training or model tuning. Given their relevance, the M-pro, spike, PLpro, and RNA polymerase genes were included in the test set [54,55,56] (Figure S2). The genes in the validation and test sets were chosen in order to have a similar number of mutations per nucleotide between them (Figures S2 and S3).

**Figure 2:**
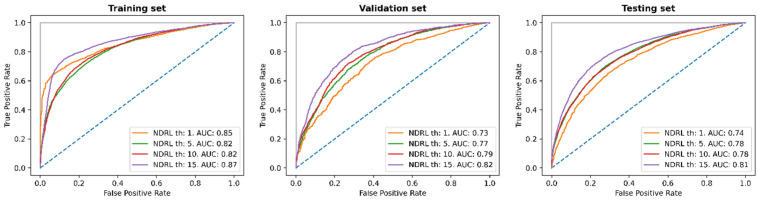
Receiver operating characteristic (ROC) curve for the testing, validation, and training set using 1, 5, 10, and 15 as thresholds for the NDRL. The blue dashed diagonal line represents how a random model would behave.

We decided to prioritize sensitivity (true positive rate) over specificity (true negative rate) in choosing the best prediction model. We chose the model that achieved the highest specificity with a minimum sensitivity of 0.85 in the validation set. As the NDLR threshold increased, the performance of the trained model on the testing, validation, and training set improved (Figure 2). This is shown by the increase in the area under the curve (AUC) of the receiver operating characteristic (ROC) curve. The AUC values for the training set were between 0.82 and 0.87 and, as expected, were higher than the values for the validation and testing sets. Interestingly, the values for the validation and testing sets were similar. The best AUC for the testing set was achieved for the NDRL threshold of 15, with an AUC of 0.81. This shows that it is possible to predict the position of recurrent SARS-CoV-2 mutations. When analyzing the model’s performance on the test set genes separately for the four genes included in this set (M-pro, spike, PLpro, and RNA polymerase), for the NDRL threshold of 15, the prediction is worse for the spike gene (with an AUC value of 0.77) (Figure S4). This is not uncommon, as mutations in the spike gene can have a high impact on the infectious power of the virus and these mutations are the most difficult to predict.

**Figure 3:**
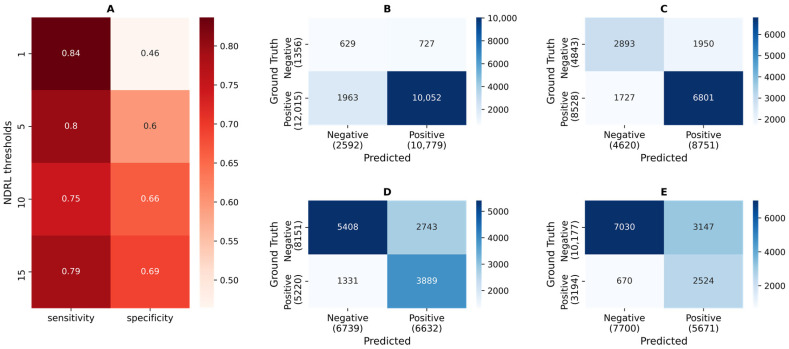
Sensitivity, specificity, and confusion matrix of the test set using the thresholds 1, 5, 10, and 15 for the NDRL. (**A**) shows sensitivity and specificity. (**B**–**E**) show, respectively, the confusion matrix using the NDRL thresholds of 1, 5, 10, and 15. The values are not normalized. Therefore the color cannot be compared between subplots. The ground truth, true categories, is placed on the left, and predicted values on the bottom. True positives are on the bottom right, and true negatives are on the top left.

Figure 3 shows the sensitivity, specificity, and confusion matrix of the test set across the four NDRL thresholds. The four predictive models showed similar sensitivity values, but as the NDRL thresholds increased, specificity also increased from 0.46 to 0.69. Confusion matrices show that when the degree of RM is low, 1 or 5 NDRL, more positions in the SARS-CoV-2 genome have an RM. In this case, predictive models perform well for predicting true positive cases but perform worse for predicting true negative cases. When the NDRL threshold increases, the number of RM decreases, but predictive models are able to predict reasonably well the positions in the genome that do or do not have an RM.

We hypothesized that some positions predicted by the model as false positives might become true positives later on. To test this hypothesis, we used model predictions trained with data from 19 April 2021 but with updated ground truth from 6 January 2022. Table 1 shows the percentage of the predicted false positives that turned into true positives and other variables for various NDRL thresholds for considering a mutation as RM in the January 2022 ground truth. We used different NDRL thresholds because the number of lineages for each mutation in the January 2022 data is three to four times higher than in the April 2021 data. The AUC and sensitivity of the RM position prediction increase as the NDRL threshold increases (Table 1). When using an NDRL threshold of 45, 17.7% of the false positives predicted for the NDRL threshold of 15 turns into true positives in January 2022. At this NDRL threshold, the AUC, sensitivity, and specificity of the RM position prediction are 0.8, 0.747, and 0.716, respectively (Table 1). All these metrics correspond to the testing set. These data confirm our hypothesis that some of our predicted false positives become true positives later on.

### 2.4. Global Feature Importance of the Prediction of Whether a Given Position in the SARS-CoV-2 Genome Will Be Affected by a Recurrent Mutation

**Figure 4:**
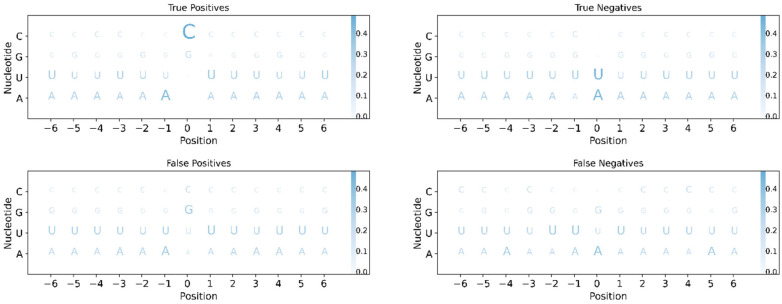
Logos of the true positives, true negatives, false positives, and false negatives when the NDRL threshold is 15. Each subplot shows the position on the horizontal axis and a row per nucleotide on the vertical axis. Each column/position for every subplot is normalized vertically, so they add up to 1. The intensity of the blue correlates with the size of each letter, so a more frequent nucleotide appears in a darker blue and a larger letter size.

Neural networks are often described as black-box models when the influence of each input variable on the success of the model is unknown. We used the Shapely Additive exPlanations (SHAP) [57] to determine the influence of each variable on whether a position in the trained model would mutate or not. The most important features are those with the highest normalized SHAP values (see Materials and methods). We analyzed four models with NDRL thresholds of 1, 5, 10, and 15 from April 2021. The nucleotide in the central position (P0) of each evaluated window of 13 positions (P-6 to P6) is the most important variable in predicting the position of the SARS-CoV-2 genome where an RM will take place (Figure S5). Other important variables are the nucleotides in other positions (e.g., P1, P-1, P2) and the in vivo and in vitro RNA SHAPE-Seq reactivity data [53]. When the NDRL threshold is higher, the most relevant variables become more important. Mainly cytosines, and to a lesser extent, guanines, are more prone to being RM (Figure 4 and Figure S6). False positives have either a guanine (35%) or a cytosine (25%), and true negatives have mainly adenine (46%) and uracil (45%) (Figure 4). Regarding the nucleotides surrounding the nucleotide that mutates, at an NDRL threshold of 15, the upstream and downstream positions (P-1 and P1, respectively) are the most relevant. In general, the other positions are of little importance (Figure 4 and Figure S6). In 44% and 27% of the true positives, there is an adenine or an uracil at P-1, and in 37% of the cases, there is an uracil at P1. This is consistent with evidence that the cytosines of the UC and AC motifs of the SARS-CoV-2 genome are preferentially deaminated by the APOBEC3A and APOBEC1 enzymes [12]. The importance given to the SHAPE-Seq reactivity comes after that of the nucleotides (Figure S5). However, the magnitude of their importance is at least five times lower. Low SHAPE-Seq reactivity values, in the range of 0 to 0.69, do not promote mutagenesis at most positions (Figure S7). However, higher SHAPE-Seq reactivity values lead to mutations (Figure S7). This analysis of the most important variables is compatible with a model that mainly predicts cytosines of the ACU pattern as RM in a region with an RNA structure that makes this cytosine more reactive. This is consistent with the SARS-CoV-2 mutational bias pattern and the preference of some host deaminases for specific sequences and RNA secondary structures [11,12,14].

### 2.5. Prediction of Whether a Given Mutation Will Be a Recurrent Mutation

We developed another machine learning method, this time to predict the NDRL in which we can find a specific mutation, i.e., whether a specific mutation will become an RM. The data were split into training, validation, and testing sets, in the same manner as described before. Similarly, the model selection was also chosen by maintaining a minimum value of 0.8 for the sensitivity in the validation set and selecting the model that achieved the highest specificity. The performance of this prediction method was similar to the previous one. The ROC-AUC of the prediction of whether a mutation will be found in more than 15 NDRLs was 0.88, 0.83, and 0.84 for, respectively, the training, validation, and testing sets (Figures S8 and S9). In the testing set, once again, the worst prediction was found in the spike gene (AUC 0.82, Figure S9). The most important variables for predicting the NDRL of a mutation were the starting nucleotide, towards which it mutates, and the in vitro SHAPE-Seq reactivity (Figure S10). For the NDRL threshold of 15, (a) the most important variable is when a nucleotide mutates into an uracil (>U at Figure S10), and (b) adenine and cytosine were the most relevant starting nucleotides (A> and C> at Figure S10). Again, this is compatible with a model that predicts the mutations C > U and A > G to be recurrent.

### 2.6. Evaluation of the Models with the Variants of Concern

A good way to test the usefulness of our predictions is to check whether our models could have predicted the mutations we found in the variants of concern (VoCs). The identification of the positions of the testing set that mutate in the Alpha, Beta, Delta, Gamma, and Omicron VoCs has an accuracy of 0.636, 0.600, 0.778, 0.80, and 0.697, respectively, when using the ground truth of January 2022 and an NDRL threshold of 45 (Table 2). The accuracy for predicting the mutations of these VoCs is 0.545, 0.40, 0.33, 0.733, 0.636, and 0.60 (Table 2).

Several mutations of the testing set from the VoC are correctly predicted by our two prediction methods (Table 3 and Table S1). This is the case for the C3267U, C3828U and G5230U mutations of the PLpro gene, the G15451A mutation of the RNA polymerase, the C21614U, C21638U, C21762U, C21846U, G21974U, G22132U, C22686U, G22813U, G22898A, C23525U, C23604A, C23664U, C23709U, G23948U, C24642U, G24914C and G25088U mutations of the spike gene. Our method predicts that the C14408U mutation, present in all VoCs and that codes for the RNA polymerase P323L shift, is an RM. As this mutation was found early in the pandemic, it is found in more than 99% of SARS-CoV-2 genomes available until January 2022. This mutation is present in all Pango lineages and therefore it is not considered to be an RM. As a result, this mutation is a false positive of our predictions. Mutations A5648C and A22812C from the VoC Gamma and U6515A, G8393A, A23055G, U23075C, A23403G, and A24424C from the VoC Omicron are true negative predictions of our position and mutation prediction models. These mutations are not recurrent because they are found in less than 45 Pango lineages. Mutations C10449A, U23599G, C23854A, and C24130A (Omicron) are true positives of the position prediction and true negatives of the mutation prediction. This means that these positions contain RMs, but the particular mutations observed in these VoCs are not recurrent. It has been described that the VoC Omicron contains many mutations not observed with a high frequency in other SARS-CoV-2 genomes [58]. Other VoC mutations were false negatives of our predictions. This is the case with the A2832G and U6954C mutations from the PLpro and the A21801C, U22679C, U22917G, G23012A, A23013C, and A23063U mutations of the spike gene. The G23012A mutation from the receptor binding domain (RBD) of the spike protein causes the AA change E484K, which reduces serum neutralization efficiency [59]. The A23063U mutation is a missense mutation present in the VoCs Alpha, Beta, Gamma, and Omicron that results in the AA substitution N501Y of the spike protein’s RBD. This substitution enhances SARS-CoV-2 infection and transmission and occurs convergently in several lineages [60]. The U22917G mutation causes the AA substitution L452R that increases spike stability, viral infectivity, and viral fusogenicity and thereby promotes viral replication [61]. Although the A23063U and U22917G mutations were present, respectively, in more than 1 million and 2 million of SARS-CoV-2 genomes available up until January 2022 and in more than 280 pangolin lineages, neither of our two prediction methods predicted these positions or mutations as recurrent. These kinds of mutations, which enhance SARS-CoV-2 infection and transmission, are the most interesting ones but the most difficult to predict because they could not be caused by host deaminases. Our current prediction models are not specifically trained to detect them. Other interesting cases are those that are false positives of our predictions. The C14408C (RNA polymerase) and C24503U (spike) mutations are found in a few SARS-CoV-2 genomes but are now in the VoC Omicron. They are false positives of our predictions because they are found in very few cases until January 2022. They could be mutations that were not observed because they have a negative impact on SARS-CoV-2, or they could be mutations that may be recurrent in the future, and it would therefore be interesting to monitor them.

### 2.7. Prediction of AA Changes Caused by Recurrent Mutations in the M-Pro and Spike Proteins

We used our model to predict whether a specific mutation is recurrent to evaluate all possible mutations in the M-pro and spike proteins. The predicted mutations obtained with the model trained with data from April 2021 produced a set of possible AAs that were compared with the AA found in the ground truths from April 2021 and January 2022. We obtained a ROC-AUC of 0.849 and 0.687 for the M-pro and spike proteins, respectively (Table 4). For this calculation, we took all AAs that were neither observed nor predicted to mutate as true negatives. The 8 and 102 AA positions for M-pro and spike proteins among the false positives of the RM prediction became true positives with the data from January 2022 (Table 4).

**Figure 5:**
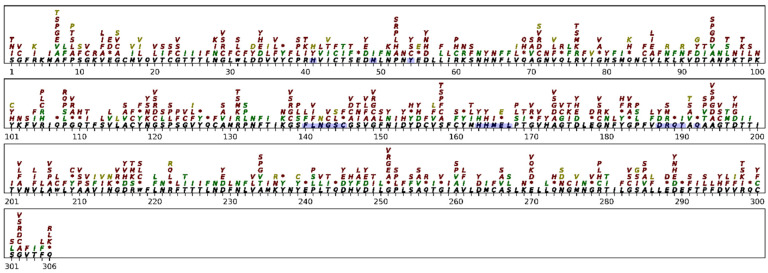
Comparison between M-pro AA changes predicted by our model and changes observed up until January 2022 (NDRL ≥ 45). The reference M-pro AA sequence is shown in black, just above the AA positions. The possible AAs produced by the predicted mutations of NDLR ≥ 15 are stacked over the reference sequence. True positives, false positives, and false negatives are shown in green, red, and dark yellow/gold, respectively. * represents a stop codon, and the same AA represents a synonymous mutation. The AAs with a blue background correspond to the subsites S1, S1′, S2, and S3.

The comparison of the predicted AA changes with the mutations observed up until January 2022 shows that more than 77% of the observed recurrent AA changes and recurrent synonymous mutations observed in the M-pro protein are well predicted by our method (Figure 5). False positives (shown in red in Figure 5) could be recurrent AA changes that will be observed in the future and are interesting to monitor. AAs that have mutated and that are thought to have other possibilities as predicted by our method, such as Ala94, Arg105, Pro108, Ala116, Ala129, Cys160, Met162, Pro168, Ala191, Ala193, Ala234, Val247, Ala260, Ala261, Arg279, and Ala285, are positions that tolerate diverse AA substitutions because they do not affect protein function [62]. Among M-pro AAs, such as Thr25, Thr26, His41, Met49, Phe140, Gly143, Cys145, His163, His164, Met165, Glu166, Pro168, His172, Asp187 and Gln189, which usually make intermolecular interactions with covalent and non-covalent inhibitors [63], only Gly143 and Pro168 show significant AAs changes caused by RM (Figure 5). In addition, in order to evaluate the performance of our prediction method, it is important to bear in mind that the false positives and false negatives of our predictions may include negatively or positively selected positions. Among the false positives, there are also deleterious mutations that are not expected to occur. Among these, there are nonsense mutations that lead to the appearance of a premature stop codon and mutations of the catalytic Cys145 and His41 [62]. The first and last AAs (a serine and a glutamine, respectively) from the M-pro are also false positives of our prediction. These two AAs are not expected to mutate because these AAs are recognized by the M-pro itself to cut the polyprotein 1a and 1b to generate the mature M-pro. Other false positives are the AAs between positions 143 and 149 (Figure 5). This region corresponds to the conserved GSCGSxG motif, which has been identified as important for initiating catalysis in SARS-CoV and MERS-CoV [64]. Among the false negatives (shown in dark yellow in Figure 5), they could be recurrent mutations. Instead of being recurrent because the host deaminases have caused them, they have been positively selected, and when they do occur, they confer a beneficial effect on virus transmission. Asn274 has several recurrent AA substitutions that our prediction method was unable to predict.

**Figure 6:**
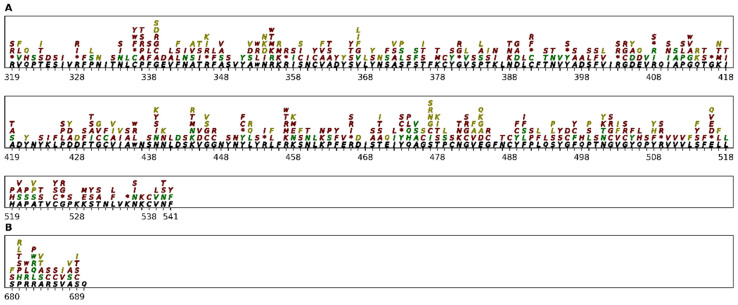
Comparison between AA changes predicted by our model and changes observed up until January 2022 from the RBD and furin cleavage site of the spike protein. The sequence from the RBD (**A**) and furin cleavage site (**B**) from the spike protein is shown in black. The AA changes predicted by our model are stacked over the reference sequence. The true positives, false positives, and false negatives are shown in green, red, and dark yellow/gold. * represents a stop codon, and the same AA represents a synonymous mutation.

The sensitivity of our predictions is only 44.2% for the spike protein, showing that the AA changes for the spike proteins are more difficult to predict (Figure S11). One of the main reasons for this low sensitivity is the high number of false negatives (Table 4). The RBD is a key functional part of the spike protein that is responsible for ACE2 binding [65]. Our prediction model showed that 46% of the recurrent AA changes and recurrent synonymous mutations observed for the RBD until January 2022 are true positives (AA in green in Figure 6A). Among the false positives (shown in red in Figure 6), there are nonsense mutations that were not expected to occur. Other false positives may include AA changes that are not observed in enough lineages to be considered RM or mutations not observed because they are deleterious. Among the false negatives (shown in dark yellow in Figure 6), there are mutations that our method had not predicted as recurrent but that gives an advantage to the virus. These include some of the mutations observed in some of the VOCs discussed earlier, such as L452R [61], E484K [59], and N501Y [60]. Another interesting region of the spike protein to be studied is the furin cleavage site, which plays a key role in the cell tropism and pathogenesis of SARS-CoV-2 [66]. This cleavage site contains the residues PRRARS at positions 681–686 of the spike protein. Figure 6B shows our mutation predictions for this region. Some of the mutations in this region are expected to be rare because they may reduce the cleavage caused by the furin protein [66]. This is the case with R682, R685, and S686. The AAs substitutions R682L and R682W are predicted by our methodology to be caused by the RM G23607T and C23606T, respectively. They are observed in a few SARS-CoV-2 lineages and are false positives of our prediction (Figure 6B). The R685C, R685S, and S686C changes are also false positives of our predictions for the same reason. R683 seems to be not so important. AA changes of R683 to other AAs, i.e., L, Q, and W, are recurrent, as our methodology correctly predicted (Figure 6B). Our methodology also correctly predicted that the P681H substitution observed in the alpha variant was caused by an RM. This substitution may slightly increase the furin cleavage, but it has no effect on viral entry or cell-cell spread [67]. However, the P681R substitution observed in the delta variant caused by the C23604G mutation is a false negative of our prediction.

## 3. Materials and Methods

We used 877,086 SARS-CoV-2 genomes from the GISAID database [44,68] available until 19 April 2021, to create the predictive model, and 4,616,059 SARS-CoV-2 genomes from 6 January 2022 to evaluate the model. Only genomes with a high coverage were considered. The NC_045512.2 genome [69] was set as a reference genome in order to align and identify mutations. The mutations, date, pangolin lineage, and genome ID were captured for each genome. Insertions and deletions were not taken into account. Only mutations from A, G, C, and U to A, G, C, and U were considered. For each mutation, we took the position and calculated the number of different pangolin lineages where this mutation was observed. We applied an algorithm to group the lineages that were linked together so that the whole group could be counted as one, thereby reducing the number of lineages for each mutation. We then calculated the NDRL.

### 3.1. NDRL Algorithm

We established a set of thresholds to define when a mutation belongs to a lineage and a group of linked lineages.

Th1: Threshold that defines when a mutation (grouped by the position that mutates) belongs to a lineage. If a mutation is present in at least th1% of the genomes that belong to that lineage, we say that it belongs to that lineage or that those mutation-genomes are related. In our calculations, we considered that a mutation belongs to a lineage if it is in at least 60% of that lineage’s genomes; therefore, Th1 is 0.6.

Th2: Threshold that defines when a mutation belongs to a group of related lineages. If a mutation belongs (marked by Th1) to at least th2% of the lineages of related lineages, we say it belongs to all those lineages for that mutation/position. In our calculations, we considered that a mutation belongs to a group of related lineages if it belongs to at least 60% of them. Thus, Th2 is 0.6 as well.

A group of related lineages is a lineage and all its descendants. For example, A.1.* means all the lineages that begin with A.1. [A.1.1, A.1.2, …, A.1.10]. When a mutation belongs to a group of related lineages, the NDRL count is equal to one for that whole group. Therefore, it is easier to count from parent to children, from a more general, bigger group to a more specific one.

For each mutation, we visited each lineage, parents first, and evaluated which complied with the Th1 and Th2 values. If the parent lineage complied, it was grouped with all its children and counted as 1. All these children were then excluded from further evaluations. If there was no group of related lineages, then the NDRL count was equivalent to the number of lineages where the mutation was present.

### 3.2. Data Set Composition

Our main focus was finding future mutations in the genes M-pro, spike, PLpro, and RNA_pol. Therefore, these genes became the test set. Among the other genes, those that have a similar length are helicase, nsp6, endoRNAse, and M. Thus, we used these for the validation set. This means the training set was composed of the remaining genes: leader, nsp2, nsp4, nsp7, nsp8, nsp9, nsp10, exonuclease, methyltransferase, ORF3a, E, ORF6, ORF7a, ORF7b, ORF8, N and ORF10. For each position in the selected genes, a window of six positions was taken on each side, before and after. Therefore, the input of the models had 13 positions of the genome, the central position being the one under analysis. A higher number of positions did not improve the performance of the trained models. A set of features were considered for each position of every window: mRNA nucleotide, RNA normalized 2′-hydroxyl acylation analyzed by primer extension (SHAPE) reactivity [53], secondary structure information calculated using Vienna RNAfold [70], and the AA to which it is going to be translated. The secondary structure information was composed using a forgi file format, and if it was connected to another nucleotide, we stated to which one it was connected. We converted the variables that did not have a numerical representation to a one-hot encoding representation. There were some missing values in the reactivity data, so we used a multivariate imputation method [71,72,73]. For the position prediction, we grouped positives and negatives into four groups. These groups contained mutations that were at least in 1, 5, 10, or 15 NDRL. The NDRL was defined using the Th1 and Th2 equal to 0.6. Therefore, when the threshold was set to 5, those positions with an NDRL lower than 5 were set as negatives and those with a higher value as positives. Therefore, each mutation with a threshold (th) of 15 was also a mutation in the group with a threshold of 10, 5, and 1. When higher NDRL values were evaluated, the performance increased, but the number of mutations decreased substantially. For the mutation prediction models, we followed the same steps but introduced a few changes. We only had three groups, NDRL 5, 10, and 15. NDRL 1 was excluded because we only worked with registered mutations. Positions with no registered mutations were not included, so it was not possible to define a negative category for the NDRL lower than 1. The other change was the addition of the nucleotide to which the position in the center of the input window would mutate.

### 3.3. Machine Learning

We used an artificial neural network (ANN) and multi-layer perceptron (MLP) architecture. To find the best hyper-parameters, such as the number of layers and neurons per layer (Table S2), we used the Scikit-Optimize library [74]. We used a search space range between 1 and 14 layers and between 1 and 2048 neurons per layer. The search space limits were set up so that it could be tested in less than a week and fit into a 12 GB GPU Memory. We used early stopping as the regularization technique. Our model selection criteria consisted in considering only models with at least 0.8 of sensitivity and the highest possible specificity. Details about the metric implementation can be found in the file model_selection_metric.py at https://github.com/bsaldivaremc2/sarscov2_rm_prediction (accessed on 27 October 2022). We also tried convolutional neural networks (CNN) and transformers [75] architectures. The metrics obtained were comparable. However, MLP training was faster than training a transformer. In order to understand the models’ feature importance, an MLP was simpler to integrate with the SHapely Additive exPlanation (SHAP) [57] library than CNNs. We also tried a non-ANN approach with TPOT [76], but the performance was worse. In addition, a similar AUC was obtained using Autokeras [77], but it lacked the flexibility to be integrated with our model selection criteria while maintaining good results and explainability. We used the mljar-supervised package [78] to generate a baseline of ensemble machine-learning models so that we could compare the performance of our models to other methods (including traditional machine learning models). A comprehensive list of the performance indicators for our chosen models and this baseline can be found in Tables S3 and S4. Our model outperforms the baseline in terms of meeting our model selection criteria (Tables S3 and S4). By using McNemar’s test [79,80], we demonstrate in Table S5 that the differences between our models and the baseline are significant. The uncertainty quality of the models, measured with the Brier score [81], is available in Table S6. To obtain the most important features, we used the SHAP values. One SHAP value was extracted per sample. Therefore, in order to obtain the general importance of a specific feature, we took the absolute value of all SHAP values and added those values to each feature (Equation (1)).


(1) $$F_{j}=\sum_{i=1}^{i=N}(V_{i}),F_{nj}=\frac{F_{j}}{\sum_{i=1}^{i=M}F_{i}}$$

*F_j_* is Importance of Feature *j*.

*V_i_* is the SHAP values of sample *i*.

*N* is the number of samples.

*F_nj_* is the normalized Importance of Feature *j*.

*M* is the number of features.

To evaluate the predictions of our models with the test set genes, we used updated data from 6 January 2022. We used this data as a new ground truth, as shown in the Results and discussion section. Nevertheless, the number of lineages and the NDRL had changed for the known mutations from 2021 (with which we trained our models). Therefore, we calculated the growth factor for these known mutations NDRL2022/NRDL2021. The majority grew by a factor of three (15% between 2.75 and 3.25, 26% between 2.5 and 3.5). So, we multiplied the NDRL threshold from 2021 by three, which gave us a correspondence of 1/3, 5/15, 10/30, and 15/45 for 2021/2022. This resulted in an NDRL threshold of 45 instead of 15.

We obtained the list of variants of concern and the mutations that define them from the WHO [82] and covariant [83] websites. For the development of the machine learning models, we used a computer with 32 CPU threads, a 12 GB GPU, and 64 GB RAM.

## 4. Conclusions

Overall, we have created a novel methodology that uses an artificial neural network capable of predicting RM in the SARS-CoV-2 genome. We have used the SARS-CoV-2 genome sequence, SHAPE-Seq reactivity values, and other variables to predict the position that mutates, the mutation that occurs, and the AA changes involved. We have validated our predictions using a test set of four genes that includes the M-pro and the spike genes, as well as using a real-case scenario such as the prediction of RM in VoCs. The model is robust enough to predict mutations in the long term, as some false positives within a limited time frame become true positives in an extended period of time. The predictive method also may be useful for finding positively and negatively selected positions in the SARS-CoV-2 genome. Among false positives, there are deleterious mutations that were not expected to occur. Among false negatives, there could be positions that have been positively selected, and when they occur, they confer a beneficial effect on virus transmission. These results can be used to find antiviral drugs that will be effective against future SARS-CoV-2 mutations.

## Figures and Tables

**Table 1:** Performance metrics of models trained with data from 19 April 2021 and evaluated with data from 6 January 2022. This table shows the metrics (ROC-AUC, sensitivity, specificity, accuracy), false positives from 2021 (fps in 2021) that turn into true positives (fps in 2021 to tps in 2022), and the proportion of this conversion (fps to tps ratio) using different NDRL thresholds for the data from 2022 (th true January 2022).

NDRL Threshold Pred 04/2021	NDRL Threshold True 01/2022	ROC-AUC	Sensitivity	Specificity	Accuracy	Fps in 2021	Fps in 2021 to Tps in 2022	Fps to Tps Ratio
15	15	0.644	0.481	0.724	0.549	3147	2119	0.673
15	30	0.728	0.597	0.743	0.671	3147	1402	0.446
15	45	0.800	0.747	0.716	0.726	3147	557	0.177
15	60	0.848	0.853	0.681	0.715	3147	99	0.031
15	75	0.873	0.910	0.655	0.691	3147	14	0.004
15	90	0.879	0.936	0.636	0.668	3147	5	0.002
15	105	0.877	0.939	0.622	0.647	3147	2	0.001
15	120	0.880	0.949	0.612	0.634	3147	2	0.001
15	135	0.883	0.953	0.606	0.625	3147	0	0

**Table 2:** Performance over variants of concern.

Position Prediction. NDRL 15/45 * (2022)					
Variant of Concern	No. of Mutations	No. of Mutations NDRL45	Accuracy	Sensitivity	Specificity
Alpha	11	8	0.636	0.750	0.333
Beta	10	8	0.600	0.625	0.500
Delta	9	7	0.778	0.857	0.500
Gamma	15	11	0.800	0.818	0.750
Omicron	33	24	0.697	0.708	0.667
Combined	61	49	0.697	0.776	0.667
Mutation Prediction. NDRL 15/45 * (2022)					
Alpha	11	8	0.545	0.500	0.667
Beta	10	8	0.400	0.375	0.500
Delta	9	7	0.333	0.286	0.500
Gamma	15	11	0.733	0.727	0.750
Omicron	33	17	0.636	0.471	0.812
Combined	61	42	0.607	0.500	0.842

* 15/45 means that the NDRL threshold of 15 was used for the prediction, but it was evaluated with the ground truth from January 2022, using an NDRL threshold of 45.

**Table 3:** Summary of some VoC predictions on position (pos) and mutation (mut). See Table S1 for a complete table.

Position	VoC *	Gene	Mutation	AA	N ^i^	Countries ^i^	NL ^i,†^	NDRL ^i^		Prediction 15/45 ^‡^	
								pos.	mut.	pos.	mut.
3267	A	Plpro	C3267U	T183I	903,866	164	246	241	238	tp	tp
21614	G	S	C21614U	L18F	167,687	145	428	399	397	tp	tp
21762	O	S	C21762U	A67V	13,723	103	244	248	244	tp	tp
23709	A	S	C23709U	T716I	904,197	167	247	234	234	tp	tp
14408	A,B,D,G,O	RNA pol	C14408U	P323L	4,577,014	193	1450	1	1	fp	fp
6515	O	Plpro	U6515A	L1266I	61	4	3	15	3	tn	tn
23403	A,B,D,G,O	S	A23403G	D614G	4,589,366	193	1460	1	1	tn	tn
24424	O	S	A24424C	Q954H	5	4	4	30	4	tn	tn
8393	O	Plpro	G8393A	A1892T	722	30	33	43	32	tn	tn
10449	O	M-pro	C10449A	P132H	1064	32	33	173	31	tp	tn
23599	O	S	U23599G	N679K	2425	38	36	138	34	tp	tn
23854	O	S	C23854A	N764K	849	27	26	200	24	tp	tn
24130	O	S	C24130A	N856K	658	32	32	314	31	tp	tn
21801	B	S	A21801C	D80A	25,012	108	88	133	84	fn	fn
22917	D	S	U22917G	L452R	2,844,958	171	321	154	137	fn	fn
23063	A,B,G,O	S	A23063U	N501Y	1,020,863	175	280	243	242	fn	fn
21801	B	S	A21801C	D80A	25,012	108	88	133	84	fn	fn

* A: Alpha, B: Beta, D: Delta, G: Gamma, and O: Omicron VoC. ^i^ On 6 January 2022. ^†^ Number of Pango lineages. ^‡^ 15/45 means that the NDRL threshold of 15 was used for the prediction, but it was evaluated with the ground truth from January 2022, using an NDRL threshold of 45. tp, fp, tn, and fn mean true positive, false positive, true negative, and false negative, respectively.

**Table 4:** Amino acid change predictions in M-pro and spike proteins.

Gene	Year ^†^	tp	fp	fn	tn	tnp	acc	spec	Sens	roc-auc
spike	2021	371	1880	471	24,032	113	0.912	0.927	0.441	0.684
	2022	473	1778	596	23,907	103	0.911	0.931	0.442	0.687
M-pro	2021	133	492	26	5775	22	0.919	0.921	0.836	0.879
	2022	141	484	41	5760	22	0.918	0.922	0.775	0.849

^†^ Date of the ground truth used to evaluate the model. 2021 means the ground truth from 19 April 2021, and 2022 means the ground truth from 6 January 2022. The columns acc, spec, sens, tp, fp, fn, tn, and tnp stand for accuracy, specificity, sensitivity, true positives, false positives, false negatives, true negatives, and true negative positions, respectively.

## Data Availability

The machine learning models and information on their use are available in the GitHub repository https://github.com/bsaldivaremc2/sarscov2_rm_prediction (accessed on 27 October 2022).

## Supplementary Materials

The following supporting information can be downloaded at: https://www.mdpi.com/article/10.3390/ijms232314683/s1.
