# Supplementary material for: Prediction of Recurrent Mutations in SARS-CoV-2 Using Artificial Neural Networks
Source: Int J Mol Sci. 2022 Nov 24;23(23):14683. doi: 10.3390/ijms232314683 (PMC9736107; doi:10.3390/ijms232314683)
Supplement: Supplementary file 1 [file ijms-23-14683-s001.zip › Supplementary_Figures and Tables_vR2.pdf]

Supplementary Information for
Prediction of recurrent mutations in SARS-CoV-2 using artificial neural networks.

Bryan Saldivar-Espinoza,[1] Guillem Macip,[1] Pol Garcia-Segura,[1] Júlia Mestres-Truyol,[1] Pere Puigbò,[2,3,4] Adrià Cereto-Massagué,[5] Gerard Pujadas,[1]* and Santiago Garcia-Vallve[1]*

[1] Departament de Bioquímica i Biotecnologia, Research group in Cheminformatics & Nutrition, Campus de Sescelades, Universitat Rovira i Virgili, 43007 Tarragona, Catalonia, Spain.
[2] Department of Biology, University of Turku, 20500 Turku, Finland.
[3] Department of Biochemistry and Biotechnology, Rovira i Virgili University, 43007 Tarragona, Catalonia, Spain.
[4] Nutrition and Health Unit, Eurecat Technology Centre of Catalonia, 43204 Reus, Catalonia, Spain
[5] EURECAT Centre Tecnològic de Catalunya, Centre for Omic Sciences (COS), Joint Unit Universitat Rovira i Virgili-EURECAT, Unique Scientific and Technical Infrastructures (ICTS)

*Correspondence: Santiago Garcia-Vallve

Email: santi.garcia-vallve@urv.cat (S.G.-V.)

This PDF file includes:

Figures S1 to S11
Tables S1 to S6

Other supplementary materials for this manuscript include the following:

Dataset S1 is in the file SI_Datasets.xlsx

Figure S1. Recurrent mutation changes at nucleotide level per Number of Distantly-Related Lineages thresholds (NDRL).

This plot shows each nucleotide change divided by the total number of mutations per NDRL thresholds. Each column adds up to 100%. The horizontal axis include in parentheses the number of total mutations per each considered threshold.

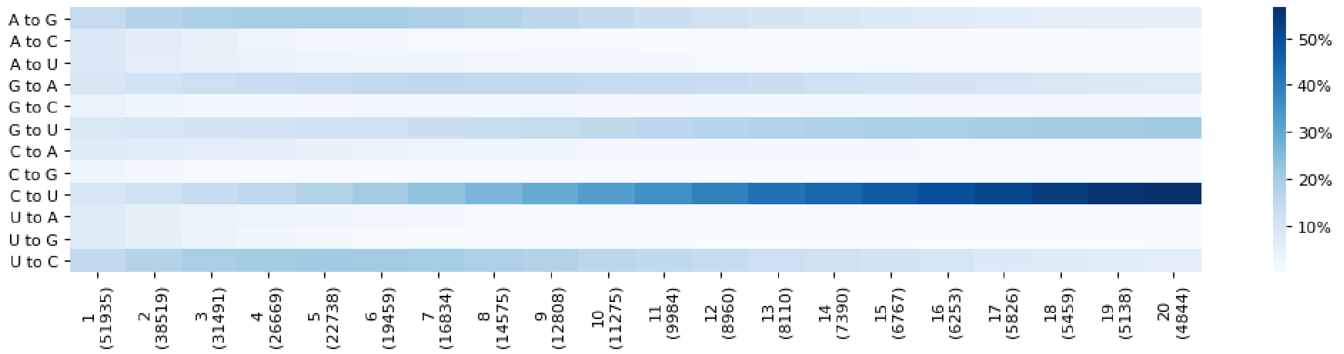

Figure S2. Number of mutations per number of nucleotides per gene across training, validation and testing set.

This plot shows the genes used in the training set (in blue), validation set (in orange) and testing set (in green). They are sorted from left to right so the gene with the highest number of mutations per nucleotide is on the left.

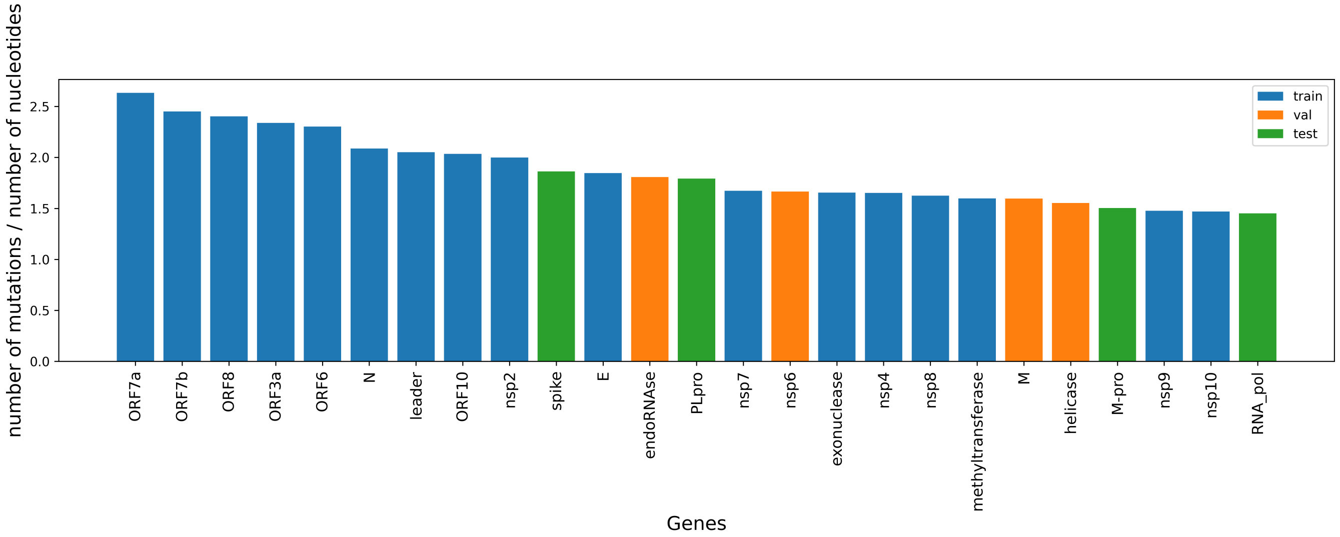

Figure S3. Mutations per set.

The columns from left to right are training, validation and testing. The plot shows the nucleotide change normalized per column, each column adds up to 100%.

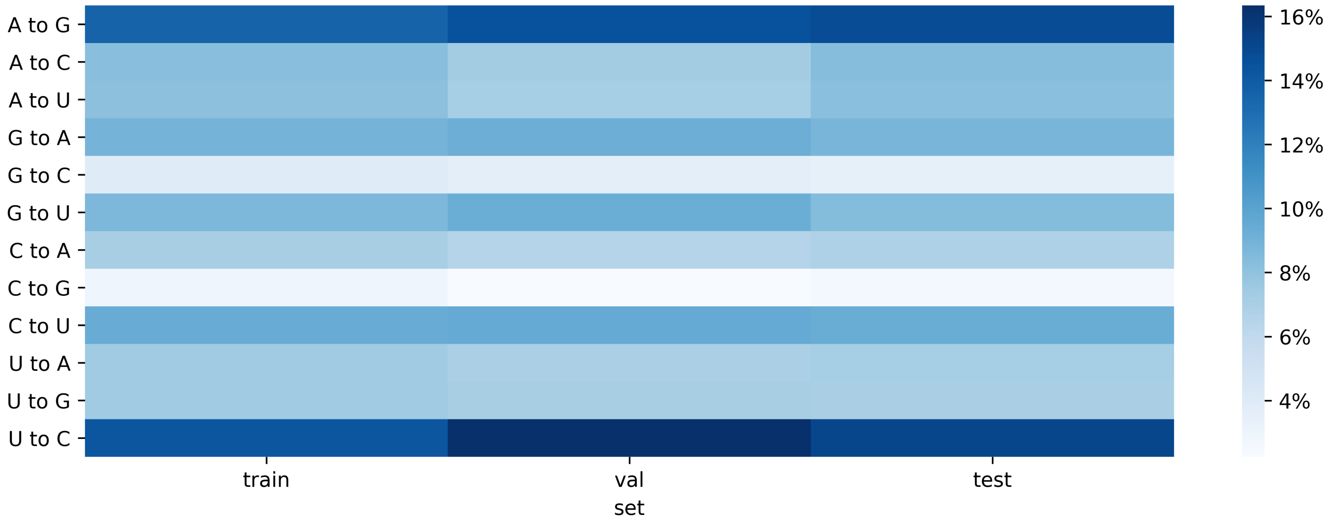

Figure S4. Receiver Operating Characteristic (ROC) curve of the testing set genes using 1 (panel A), 5 (panel B), 10 (panel C) and 15 (panel D) as thresholds for the NDRL.

The subplots A, B, C and D correspond, respectively, to the NDRL *thresholds of 1, 5, 10 and 15. Orange, green, red and purple lines correspond, respectively, to the Mpro, spike, PLpro and RNA_pol genes. The horizontal axis corresponds to the False Positive rate and the vertical axis to the True Positive rate. The values for the area under the curve (AUC) for a perfect prediction and for a random prediction are 1.0 and 0.5 respectively. The AUC for the genes M-pro, Spike, PLpro, RNA_pol are 0.73, 0.73, 0.76, 0.72 with the threshold 1; 0.79, 0.76, 0.77 and 0.8 with the threshold 5, 0.79, 0.71, 0.78, and 0.8 with the threshold 10 and 0.82, 0.75, 0.83 and 0.82 with the threshold 15.*

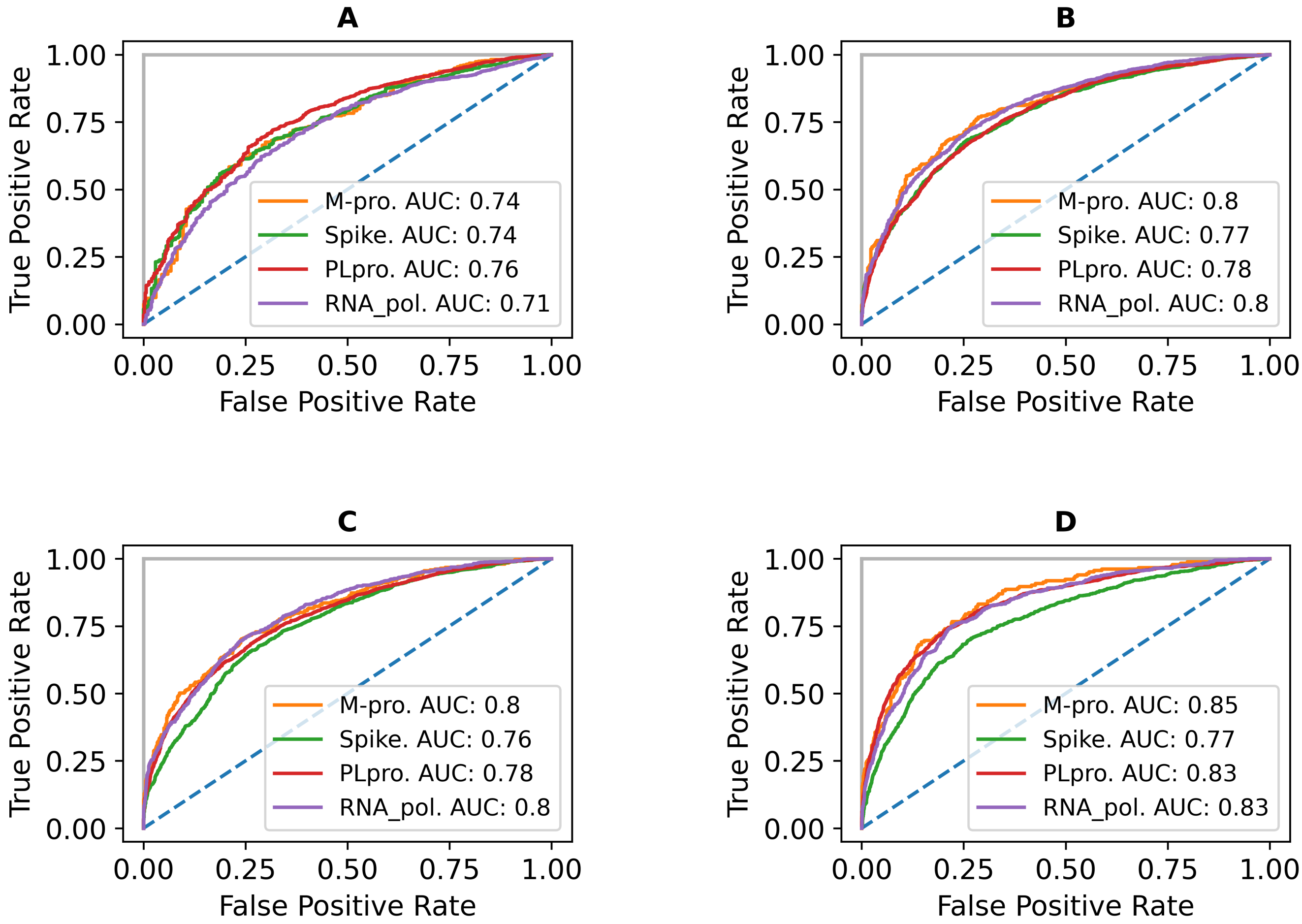

Figure S5. Top 10 most important variables in predicting the position of the SARS-CoV-2 genome where a RM will take place. Calculated across 4 NDRL **thresholds on the test set according to the SHAP values extracted from the model.**

In this Figure the most important variables appear on the top, a priority of 1 means more important and 10 less important. On the horizontal axis, from left to right the degree (threshold) of the recurrent mutation increases. A dark blue color means more important and a lighter blue less important. Each column was normalized among all the variables before cutting the top 10. The procedure to calculate this values is present in the section ***Materials and methods****.*

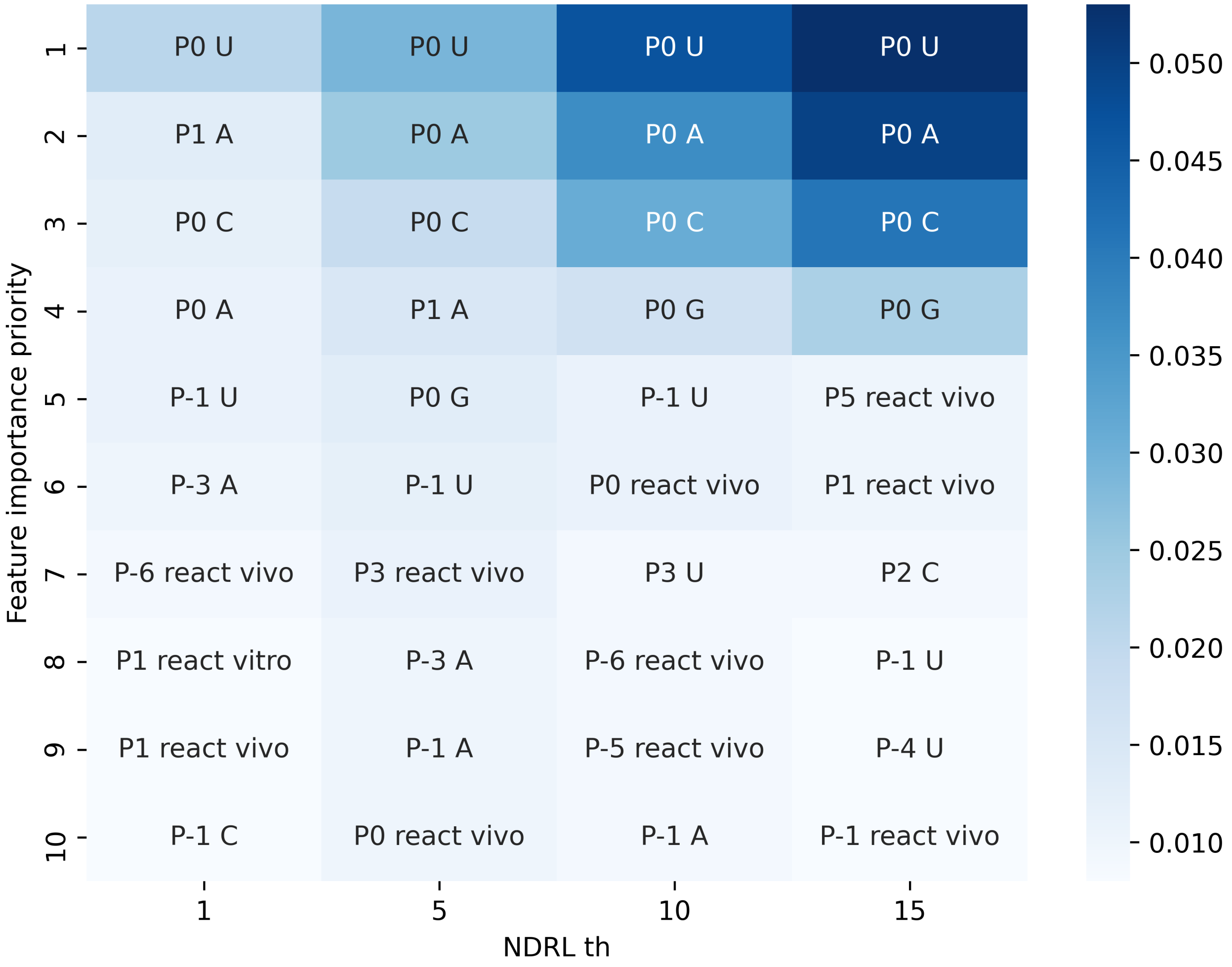

Figure S6. Mutation promoters or inhibitors' nucleotides, by position and NDRL thresholds on the test set according to the model SHAP values.

The subplots A, B, C and D correspond to the feature importance across the thresholds 1, 5, 10 and 15 of the NDRL *respectively. The values are normalized over the maximum absolute value across all the variables' SHAP values. Each box takes a positive value if that variable promotes mutations or otherwise takes a negative value.*

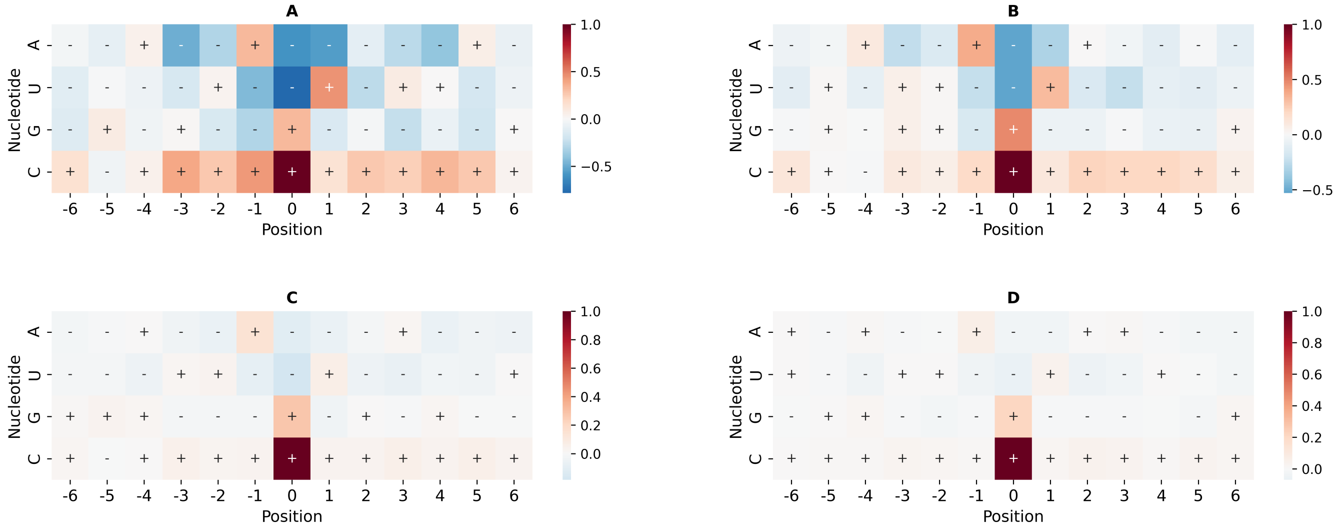

Figure S7. Mutation promoters or inhibitors' RNA normalized in vivo reactivity intervals, by position and NDRL thresholds on the test set according to the model SHAP values.

The subplots A, B, C and D correspond to the feature importance across the thresholds 1, 5, 10 and 15 of the NDRL respectively. The values are normalized over the maximum absolute value across all the variables' SHAP values. Each box takes a positive value if that variable promotes mutations or otherwise takes a negative value.

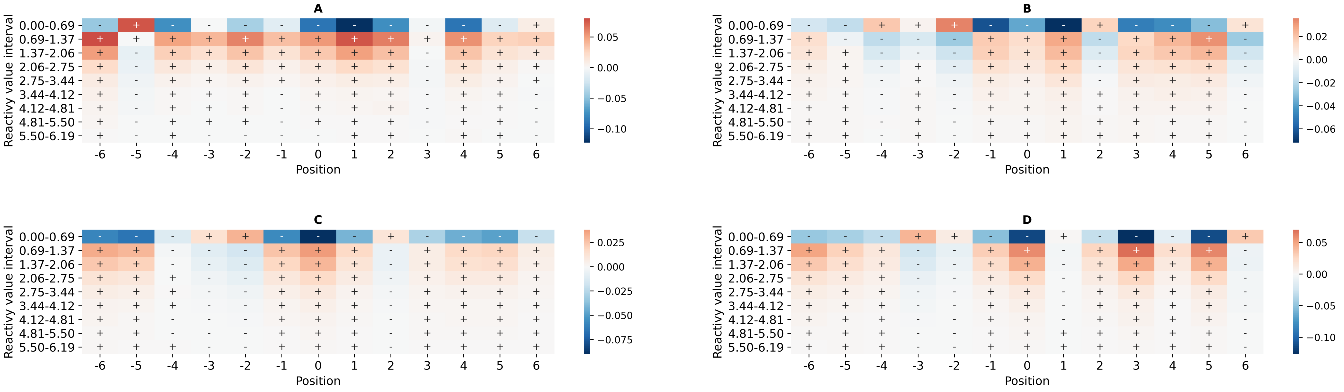

Figure S8. Receiver Operating Characteristic (ROC) curve for the testing, validation and training set using 5, 10 and 15 as thresholds for the Mutations NDRL.

The orange, green and red lines correspond to the NDRL 5, 10 and 15 thresholds. The blue dashed line in the diagonal represents a random prediction score.

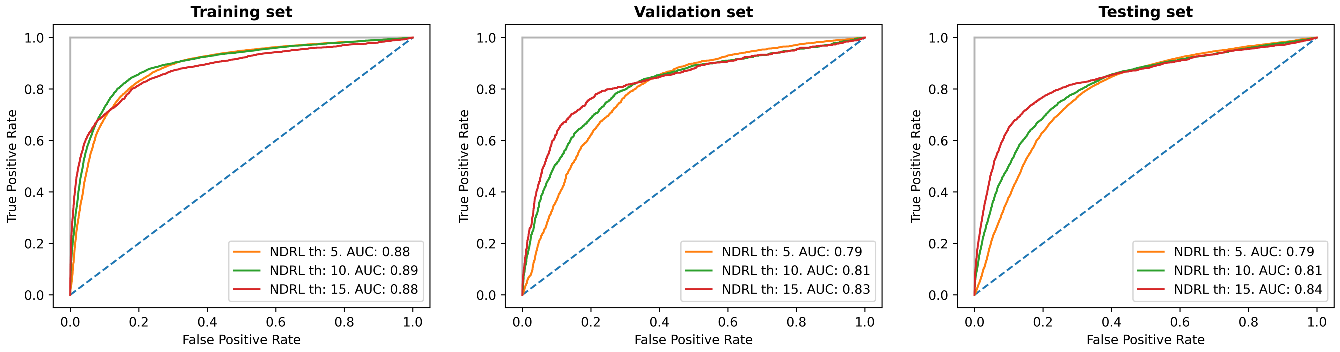

Figure S9. Receiver Operating Characteristic (ROC) curve of the testing set genes using 5, 10 and 15 as thresholds for the Mutations NDRL. *Subplots A, B and C correspond to the NDRL 5, 10 and 15 thresholds. The orange, green, red and purple lines correspond to the genes M-pro, Spike (S), PLpro and RNA_pol. The blue dashed line in the diagonal represents a random prediction score.*

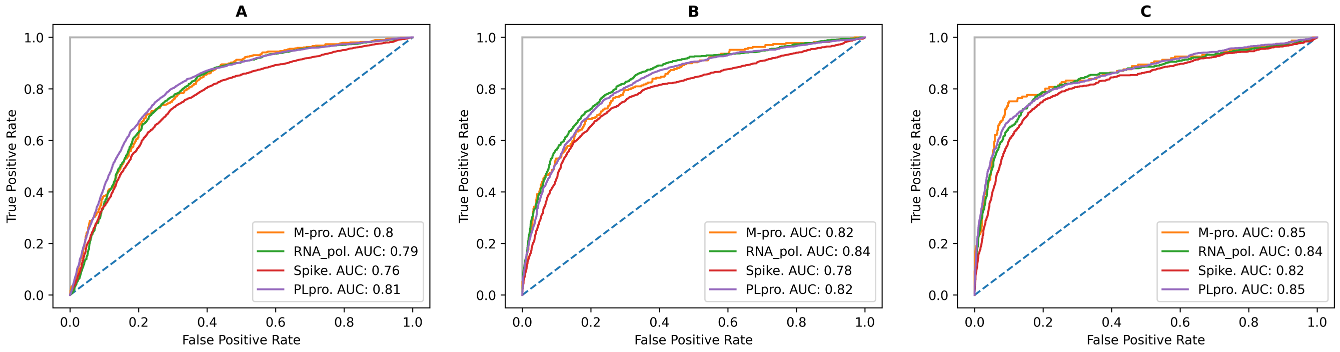

Figure S10. Top 10 most important variables in predicting the NDRL of a mutation of the SARS-CoV-2. Calculated across 3 NDRL thresholds on the test set according to the SHAP values extracted from the model.

In this Figure the most important variables appear on the top, a priority of 1 means more important and 10 less important. On the horizontal axis, from left to right the degree (threshold) of the recurrent mutation increases. A dark blue color means more important and a lighter blue less important. Each column was normalized among all the variables before cutting the top 10.The procedure to calculate this values is present in the section ***Materials and methods****.*

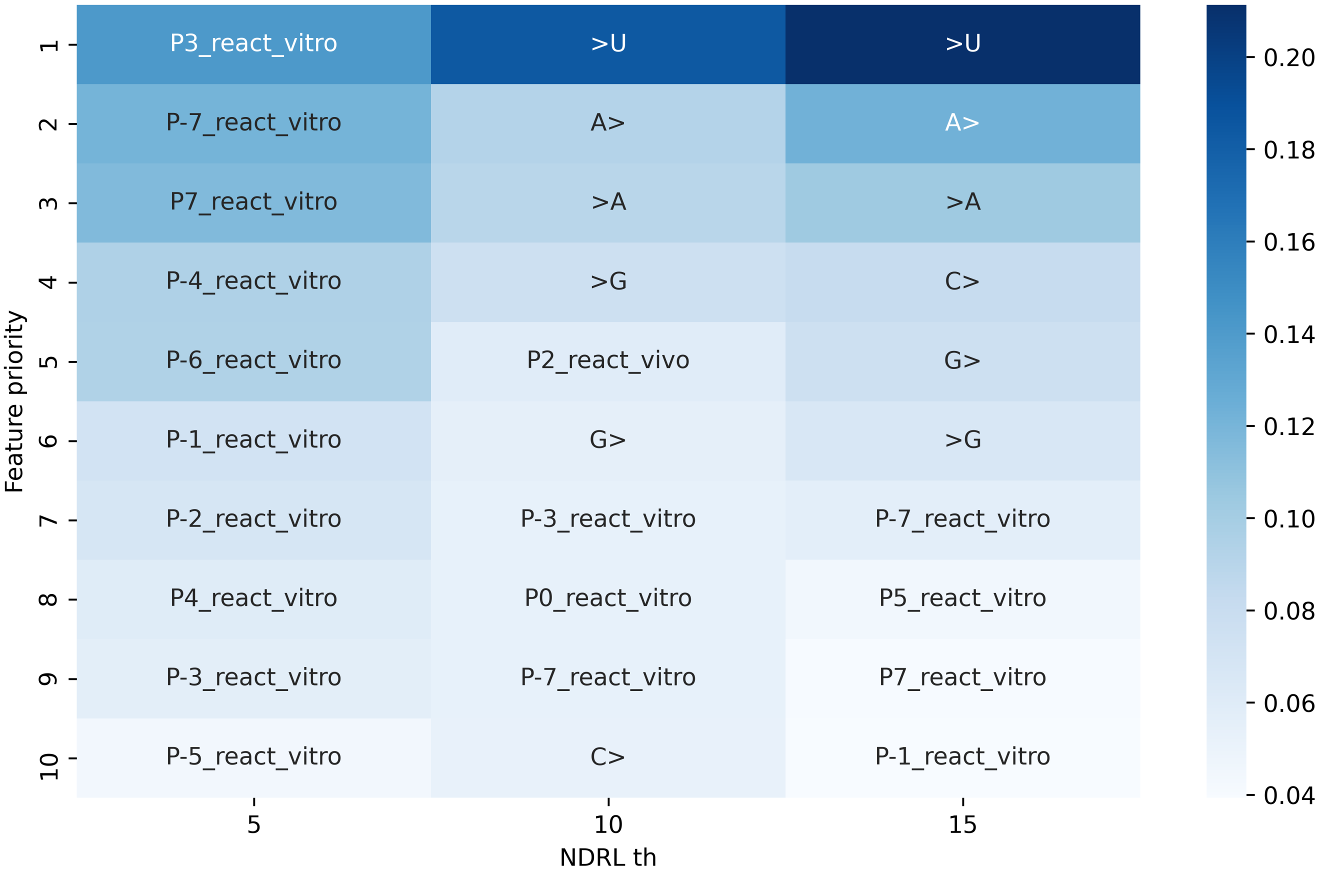

Figure S11. Spike Predicted amino acid changes, ground truth from 2022, NDRL 15.

*This Figure shows the reference Spike protein amino acid sequence in black, in the first line of each subplot. Each subplot contains the sequence split into a maximum number of 100 residues. The possible amino acids (AA) produced by the predicted mutations, of NDRL≥15, are stacked over the reference sequence. True positives, false positives and false negatives are shown in green, red and dark yellow/gold, respectively. A start * represents a stop codon and a same AA represents a synonym mutation.*

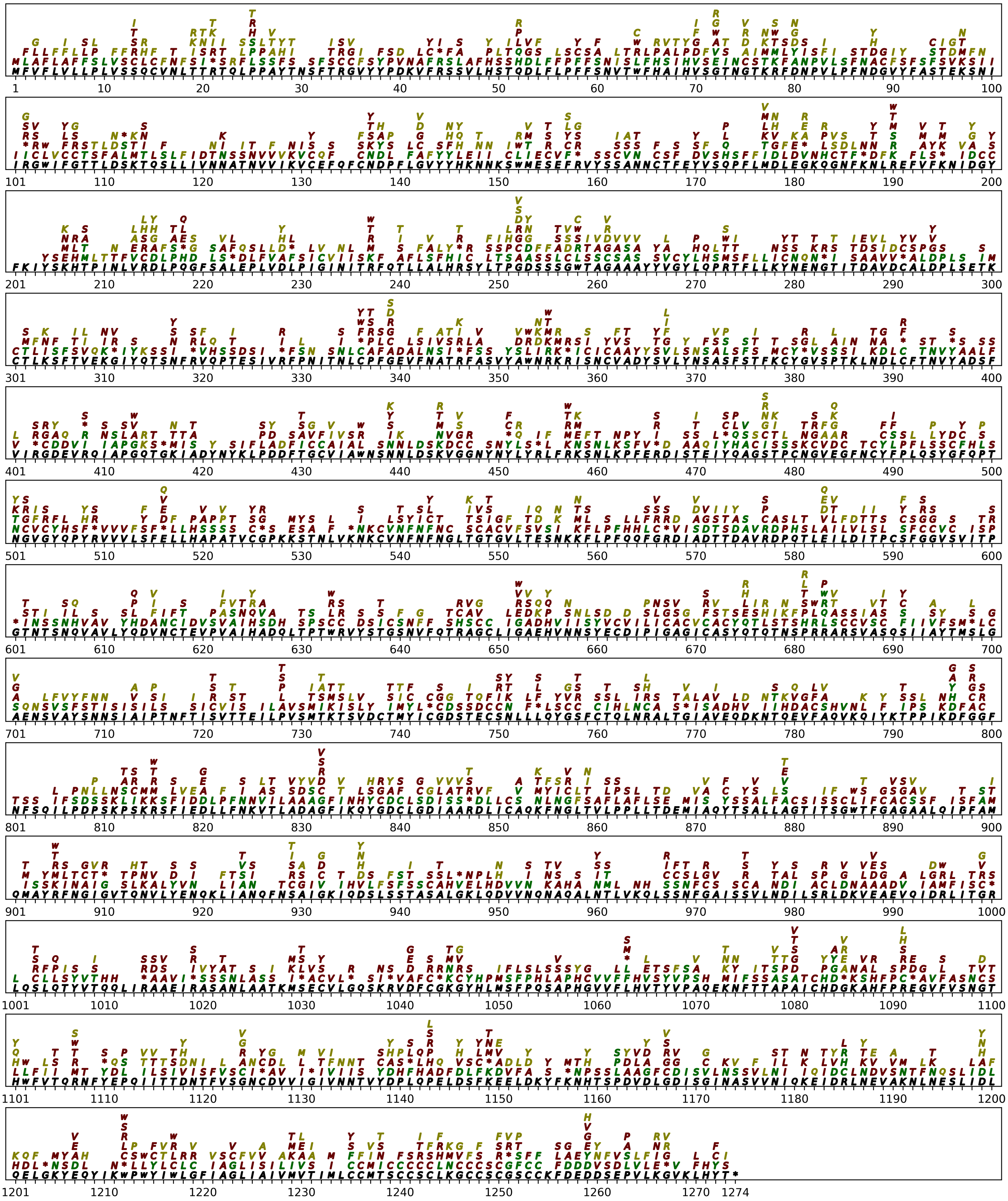

Table S1. VoC mutations of the testing set.

Position	VOC	Gene	Mutation	AA	N[i]	Countries[i]	NL[i †]	NDRL[i †]		Prediction position[‡]				Prediction mutation[‡]		
								position	mutation	1/3	5/15	10/30	15/45	5/15	10/30	15/45
2832	omicron	nsp3	A2832G	K38R	1331	39	47	48	46	tp	tp	fn	fn	tp	tp	fn
3267	alpha	nsp3	C3267U	T183I	903,866	164	246	241	238	tp	tp	tp	tp	tp	tp	tp
3828	gamma	nsp3	C3828U	S370L	90,140	100	219	198	197	tp	tp	tp	tp	tp	tp	tp
5230	beta	nsp3	G5230U	K837N	30,479	120	237	240	233	tp	tp	tp	tp	tp	tp	tp
5388	alpha	nsp3	C5388A	A890D	899,293	163	116	149	108	tp	tp	tp	tp	tp	fn	fn
5648	gamma	nsp3	A5648C	K977Q	84,776	80	60	43	39	fn	fn	fn	tn	fn	fn	tn
6515	omicron	nsp3	U6515A	L1266I	61	4	3	15	3	tp	fn	tn	tn	tn	tn	tn
6954	alpha	nsp3	U6954C	I1412T	896,419	163	159	152	151	tp	tp	tp	fn	fn	fn	fn
8393	omicron	nsp3	G8393A	A1892T	722	30	33	43	32	tp	tp	fn	tn	tp	tp	tn
10323	beta	M-pro	A10323G	K90R	96,647	157	519	514	514	tp	tp	fn	tp	tp	tp	fn
10449	omicron	M-pro	C10449A	P132H	1064	32	33	173	31	tp	tp	tp	tp	fn	fn	tn
14408	omicron gamma delta beta alpha	RNA_pol	C14408U	P323L	4,577,014	193	1450	1	1	fp	fp	fp	fp	fp	fp	fp
15451	delta	RNA_pol	G15451A	G671S	2,769,305	167	302	130	124	tp	tp	tp	tp	tp	tp	tp
21614	gamma	S	C21614U	L18F	167,687	145	428	399	397	tp	tp	tp	tp	tp	tp	tp
21618	delta	S	C21618G	T19R	2,779,017	167	237	128	58	tp	tp	tp	tp	fn	fn	fn
21621	gamma	S	C21621A	T20N	83,978	84	74	223	52	tp	tp	tp	tp	fn	fn	fn
21638	gamma	S	C21638U	P26S	94,133	109	238	222	216	tp	tp	tp	tp	tp	tp	tp
21762	omicron	S	C21762U	A67V	13,723	103	244	248	244	tp	tp	tp	tp	tp	tp	tp
21801	beta	S	A21801C	D80A	25,012	108	88	133	84	tp	tp	fn	fn	fn	fn	fn
21846	omicron	S	C21846U	T95I	1,253,114	160	452	427	420	tp	tp	tp	tp	tp	tp	tp
21974	gamma	S	G21974U	D138Y	90,868	112	261	290	239	tp	fn	tp	tp	tp	tp	tp
21995	omicron	S	U21995G	Y145D	90	8	18	110	18	tp	fn	fn	fn	fn	tn	tn
22034	delta	S	A22034G	R158G	4072	40	124	127	124	tp	fn	tp	tp	fn	tp	fn
22132	gamma	S	G22132U	R190S	83,999	91	80	110	59	tp	tp	tp	tp	tp	tp	tp
22206	beta	S	A22206G	D215G	25,381	115	138	142	134	tp	tp	tp	tp	tp	tp	fn
22578	omicron	S	G22578A	G339D	1130	44	64	69	62	tp	fn	tp	tp	tp	tp	fn
22679	omicron	S	U22679C	S373P	1003	38	58	59	56	tp	fn	fn	fn	tp	fn	fn
22686	omicron	S	C22686U	S375F	888	32	47	46	45	tp	tp	tp	tp	tp	tp	tp
22812	gamma	S	A22812C	K417T	81,007	80	36	32	15	tp	tp	fn	tn	fn	tn	tn
22813	omicron beta	S	G22813U	K417N	29,436	116	131	144	125	tp	tp	tp	tp	tp	tp	tp
22882	omicron	S	U22882G	N440K	4754	60	57	91	54	tp	tp	tp	tp	fn	fn	fn
22898	omicron	S	G22898A	G446S	1194	53	88	92	88	tp	fn	tp	tp	tp	fn	tp

22917	delta	S	U22917G	L452R	2,844,958	171	321	154	137	fn	fn	fn	fn	fn	fn	fn
22992	omicron	S	G22992A	S477N	49,484	109	235	276	205	tp	tp	tp	tp	tp	fn	fn
22995	omicron delta	S	C22995A	T478K	2,802,366	168	270	123	91	tp	tp	tp	tp	fn	fn	fn
23012	gamma beta	S	G23012A	E484K	160,657	152	306	344	269	fn	fn	fn	fn	tp	tp	fn
23013	omicron	S	A23013C	E484A	2057	58	87	118	85	tp	tp	fn	fn	fn	fn	fn
23040	omicron	S	A23040G	Q493R	899	33	37	70	35	tp	fn	fn	fn	tp	fn	tn
23048	omicron	S	G23048A	G496S	894	37	27	31	27	tp	tp	tp	fp	tp	tn	tn
23055	omicron	S	A23055G	Q498R	793	29	38	37	36	fn	tp	tp	tn	tp	fn	tn
23063	omicron gamma beta alpha	S	A23063U	N501Y	1,020,863	175	280	243	242	tp	fn	fn	fn	fn	fn	fn
23075	omicron	S	U23075C	Y505H	770	33	23	21	21	tp	fn	tn	tn	tp	fp	tn
23202	omicron	S	C23202A	T547K	1082	43	81	251	80	tp	tp	tp	tp	fn	fn	fn
23271	alpha	S	C23271A	A570D	898,365	163	94	149	86	tp	tp	tp	tp	fn	fn	fn
23403	omicron gamma delta beta alpha	S	A23403G	D614G	4,589,366	193	1460	1	1	fp	tn	tn	tn	fp	fp	tn
23525	omicron gamma	S	C23525U	H655Y	92,088	130	326	299	299	tp	tp	tp	tp	tp	tp	tp
23599	omicron	S	U23599G	N679K	2425	38	36	138	34	tp	tp	tp	tp	fn	fn	tn
23604	omicron delta alpha	S	C23604A	P681H	946,888	169	309	278	290	tp	tp	tp	tp	fn	fn	tp
23664	beta	S	C23664U	A701V	53,917	128	188	184	184	tp	tp	tp	tp	tp	tp	tp
23709	alpha	S	C23709U	T716I	904,197	167	247	234	234	tp	tp	tp	tp	tp	tp	tp
23854	omicron	S	C23854A	N764K	849	27	26	200	24	tp	tp	tp	tp	fn	tn	tn
23948	omicron	S	G23948U	D796Y	3967	74	183	216	181	tp	tp	tp	tp	tp	tp	tp
24130	omicron	S	C24130A	N856K	658	32	32	314	31	tp	tp	tp	tp	fn	tp	tn
24410	delta	S	G24410A	D950N	2,689,287	169	252	107	72	tp	tp	tp	tp	fn	fn	fn
24424	omicron	S	A24424C	Q954H	5	4	4	30	4	tp	fn	fn	tn	tn	tn	tn
24469	omicron	S	U24469G	N969K	8	4	8	73	8	tp	tp	tp	fn	tn	tn	fp
24503	omicron	S	C24503U	L981F	626	28	23	31	22	tp	tp	tp	fp	tp	fp	fp
24506	alpha	S	U24506G	S982A	898,085	163	48	40	40	tp	fn	tp	fp	fn	fn	tn
24642	gamma	S	C24642U	T1027I	91,978	106	210	193	187	tp	tp	tp	tp	tp	tp	tp
24914	alpha	S	G24914C	D1118H	896,647	163	124	194	116	tp	tp	tp	tp	fn	tp	tp
25088	gamma	S	G25088U	V1176F	98,400	111	193	169	166	tp	tp	tp	tp	tp	tp	tp

[i] *On January 6, 2022*

[†] *Number of Pango lineages*

[‡] *15/45 means that the NDRL threshold of 15 was used for the prediction, but it was evaluated with the ground truth from January 2022, using a NDRL threshold of 45. tp, fp, tn and fn mean true positive, false positive, true negative and false negative, respectively.*

Table S2. Number of neurons per layer for each model (Position prediction)

Threshold	L1	L2	L3	L4	L5	L6	L7	L8
1	1272	1963	866	451	1263	427	1764	1136
5	1927	1962	1236	2043	451	292	2048	882
10	530	2048	454	839	1508	1427	2048	219
15	1493	1676	588	413	1226	451	63	887

Table S3. Model's performance metrics - Position prediction

**Our selected model**													
th	set	sens	spec	auc	acc	tp	tn	fp	fn	Static sens	Raw static sens	Log loss	brier score
1	train	0.84	0.62	0.73	0.82	8929	510	309	1763	0.79	1.42	6.22	0.18
1	val	0.84	0.46	0.65	0.80	3260	223	260	637	0.70	1.26	7.07	0.20
1	test	0.84	0.46	0.65	0.80	10,052	629	727	1963	0.70	1.26	6.95	0.20
5	train	0.82	0.64	0.73	0.77	6786	2071	1186	1468	0.80	1.44	7.96	0.23
5	val	0.81	0.58	0.70	0.72	2188	982	697	513	0.77	1.38	9.54	0.28
5	test	0.80	0.60	0.70	0.73	6801	2893	1950	1727	0.44	0.80	9.50	0.27
10	train	0.80	0.69	0.74	0.74	4365	4138	1902	1106	0.44	0.80	9.03	0.26
10	val	0.79	0.63	0.71	0.69	1277	1752	1019	332	0.44	0.79	10.65	0.31
10	test	0.75	0.66	0.70	0.70	3889	5408	2743	1331	0.41	0.75	10.52	0.30
15	train	0.83	0.73	0.78	0.76	3013	5787	2105	606	0.85	1.53	8.13	0.24
15	val	0.82	0.68	0.75	0.71	829	2299	1068	184	0.82	1.48	9.87	0.29
15	test	0.79	0.69	0.74	0.71	2524	7030	3147	670	0.44	0.79	9.86	0.29
**Baseline with mljar-supervised, compete mode**													
th	set	sens	spec	auc	acc	tp	tn	fp	fn	Static sens	Raw static sens	Log loss	brier score
1	train	1.00	0.01	0.51	0.93	10,692	11	808	0	0.45	0.81	2.42	0.07
1	val	1.00	0.00	0.50	0.89	3897	0	483	0	0.44	0.80	3.81	0.11
1	test	1.00	0.00	0.50	0.90	12,015	2	1354	0	0.45	0.80	3.50	0.10
5	train	0.83	0.53	0.68	0.74	6833	1733	1524	1421	0.74	1.33	8.84	0.26
5	val	0.85	0.52	0.69	0.73	7291	2507	2336	1237	0.73	1.32	9.23	0.27
5	test	0.85	0.52	0.69	0.73	7291	2507	2336	1237	0.73	1.32	9.23	0.27
10	train	0.62	0.80	0.71	0.71	3377	4851	1189	2094	0.34	0.62	9.85	0.29
10	val	0.66	0.78	0.72	0.74	1057	2175	596	552	0.36	0.66	9.05	0.26
10	test	0.65	0.79	0.72	0.73	3367	6444	1707	1853	0.36	0.65	9.20	0.27

15	train	0.43	0.95	0.69	0.79	1574	7504	388	2045	0.24	0.43	7.30	0.21
15	val	0.43	0.95	0.69	0.79	1574	7504	388	2045	0.24	0.43	7.30	0.21
15	test	0.45	0.94	0.69	0.82	1425	9567	610	1769	0.25	0.45	6.15	0.18

***Th**: recurrent mutation threshold. **Sens**: sensitivity. **Spec**: specificity. **Auc**: area under the curve, **Acc**: accuracy. **Tp**: true positives. **Tn**: true negatives. **Fp**: false positives. **Fn**: false negatives. **Static sens**: Model selection metric, does not count spec while sens is lower than 0.8. Afterwards, spec is added to the maximum allowed sens 0.8. Value between 0 and 1, 1 better. **raw static sens**: Same as static sens, but no normalization. If lower than 0.8 only sens was considered. Maximum value is 1.8. **log loss**: logistic loss sum. **brier score**: uncertainty quality measurement.*

Table S4. Model's performance metrics - Mutation prediction

th	set	sens	spec	auc	acc	tp	tn	fp	fn	static sens	Raw static sens	Log loss	brier score
**Our selected model**													
5	train	0.81	0.80	0.81	0.80	8135	9343	2397	1852	0.89	1.60	6.75	0.20
5	val	0.80	0.63	0.72	0.71	2450	2623	1518	594	0.80	1.43	10.15	0.29
5	test	0.78	0.66	0.72	0.71	7582	8750	4563	2116	0.43	0.78	10.03	0.29
10	train	0.87	0.77	0.82	0.79	4471	12,713	3852	691	0.87	1.57	7.22	0.21
10	val	0.80	0.66	0.73	0.69	1172	3778	1950	285	0.81	1.46	10.74	0.31
10	test	0.79	0.67	0.73	0.70	3670	12,366	5993	982	0.44	0.79	10.47	0.30
15	train	0.83	0.76	0.79	0.77	2609	14,183	4384	551	0.87	1.56	7.85	0.23
15	val	0.80	0.70	0.75	0.71	712	4419	1881	173	0.83	1.50	9.87	0.29
15	test	0.80	0.72	0.76	0.73	2186	14,571	5720	534	0.84	1.52	9.39	0.27
**Baseline with mljar-supervised, compete mode**													
th	set	sens	spec	auc	acc	tp	tn	fp	fn	Static sens	Raw static sens	Log loss	brier score
5	train	0.76	0.83	0.79	0.80	7582	9732	2008	2405	0.42	0.76	7.02	0.20
5	val	0.80	0.76	0.78	0.78	2431	3154	987	613	0.44	0.80	7.69	0.22
5	test	0.77	0.78	0.77	0.77	7453	10,361	2952	2245	0.43	0.77	7.80	0.23
10	train	0.48	0.96	0.72	0.85	2497	15890	675	2665	0.27	0.48	5.31	0.15
10	val	0.49	0.94	0.72	0.85	720	5374	354	737	0.27	0.49	5.24	0.15
10	test	0.51	0.94	0.73	0.86	2371	17,348	1011	2281	0.28	0.51	4.94	0.14
15	train	0.51	0.96	0.74	0.90	1626	17,905	662	1534	0.29	0.51	3.49	0.10
15	val	0.55	0.91	0.73	0.87	491	5739	561	394	0.31	0.55	4.59	0.13
15	test	0.55	0.95	0.75	0.91	1491	19,357	934	1229	0.30	0.55	3.25	0.09

***Th**: recurrent mutation threshold. **Sens**: sensitivity. **Spec**: specificity. **Auc**: area under the curve, **Acc**: accuracy. **Tp**: true positives. **Tn**: true negatives. **Fp**: false positives. **Fn**: false negatives. **Static sens**: Model selection metric, does not count spec while sens is lower than 0.8. Afterwards, spec is added to the maximum allowed sens 0.8. Value between 0 and 1, 1 better. **raw static sens**: Same as static sens, but no normalization. If lower than 0.8 only sens was considered. Maximum value is 1.8. **log loss**: logistic loss sum. **brier score**: uncertainty quality measurement.*

***Table S5. McNemar's test between our models and baseline models with mljar-supervised*.**

**Position prediction**					
th	set	chi2	p<0.05	p value round	p value
1	train	705.203	True	0.000000	2.209E-155
1	val	198.336	True	0.000000	4.818E-45
1	test	688.118	True	0.000000	1.147E-151
5	train	38.141	True	0.000000	6.582E-10
5	val	0.059	False	0.807799	8.078E-1
5	test	4.006	True	0.045327	4.534E-2
10	train	29.710	True	0.000000	5.018E-08
10	val	44.497	True	0.000000	2.547E-11
10	test	100.523	True	0.000000	1.170E-23
15	train	23.993	True	0.000001	9.669E-07
15	val	164.567	True	0.000000	1.137E-37
15	test	560.524	True	0.000000	6.467E-124
**Mutation prediction**					
th	set	chi2	p<0.05	p value round	p value
5	train	6.933	True	0.008460	8.4598E-3
5	val	188.671	True	0.000000	6.199E-43
5	test	512.707	True	0.000000	1.633E-113
10	train	263.506	True	0.000000	2.953E-59
10	val	603.720	True	0.000000	2.598E-133
10	test	2031.030	True	0.000000	0
15	train	1559.527	True	0.000000	0
15	val	763.524	True	0.000000	4.601E-168
15	test	3001.633	True	0.000000	0

Table S6. Model's uncertainty quality. Brier score.

**Position prediction**			
th	train	val	test
1	0.131931	0.159333	0.155308
5	0.172322	0.207497	0.203409
10	0.204331	0.246146	0.243646
15	0.202455	0.250927	0.249452
**Mutation prediction**			
th	train	val	test
5	0.248211	0.248999	0.248900
10	0.249022	0.249823	0.249600
15	0.249590	0.250531	0.250194

The brier score goes from 0 to 1. The lower the better.
